# Supplementary material for: A Phase-I pharmacokinetic, safety and food-effect study on flubentylosin, a novel analog of Tylosin-A having potent anti-Wolbachia and antifilarial activity
Source: PLoS Negl Trop Dis. 2023 Jul 10;17(7):e0011392. doi: 10.1371/journal.pntd.0011392 (PMC10368248; doi:10.1371/journal.pntd.0011392)
Supplement: Attachment — Submitted filename: Reviewer comments_Plos.doc [file pntd.0011392.s002.doc]

Dear Editor,

I have reviewed the manuscript PNTD-D-22-00302, entitled “A Phase-I pharmacokinetic, safety and food effect 1 study on flubentylosin, a novel analog 2 of Tylosin-A having potent anti-*Wolbachia* activity and antifilarial activity” submitted to PLOS Neglected Tropical Diseases as a full-length article. Though the content is worth to be published, in my opinion this paper needs major revision for publication in PLOS Neglected Tropical Diseases.

Kind regards,

Ranju Manoj

_____________________________

Ranju Manoj, DVM, MVSc., PhD

Post-doctoral associate and resident in Veterinary Parasitology

Department of Population Medicine and Diagnostics

AHDC, Cornell University,

Ithaca, New York

Tel +1 6465207636

e.mail: rm846@cornell.edu

e.mail: drrranjuvet@gmail.com

**COMMENTS TO THE AUTHORS**

The manuscript PNTD-D-22-00302, entitled “A Phase-I pharmacokinetic, safety and food effect 1 study on flubentylosin, a novel analog 2 of Tylosin-A having potent anti-*Wolbachia* activity and antifilarial activity” is a Phase-I pharmacokinetic, safety and food-effect study on single and multiple ascending doses of the macrolide antibacterial drug, flubentylosin (ABBV-4083), targeting filarial endosymbiont, *Wolbachia* for the effective control of filariasis and onchocerciasis.

Here in this article, authors are assessing the safety and pharmacokinetics of flubentylosin in healthy subjects, to identify a dose and dosing regimen for the future studies. In general, the introduction section should include the role of *Wolbachia* in filarids and its imunopathological effects in filariasis. Here you are targeting *Wolbachia* for the treatment and hence more importance should be given for this endosymbiont. Discussion has to be rewritten. Avoid the repetition of results in discussion and discuss your results in detail. References has to be uniformly formatted. Please follow the authors guidelines of the journal while formatting. Please recheck the text carefully for grammatical and formatting mistakes. Please see the attached PDF of the manuscript for my detailed comments for the authors.
