# Supplementary material for: A Phase-I pharmacokinetic, safety and food-effect study on flubentylosin, a novel analog of Tylosin-A having potent anti-Wolbachia and antifilarial activity
Source: PLoS Negl Trop Dis. 2023 Jul 10;17(7):e0011392. doi: 10.1371/journal.pntd.0011392 (PMC10368248; doi:10.1371/journal.pntd.0011392)
Supplement: Attachment — Submitted filename: Responses to Reviewers_GS_KDP.docx [file pntd.0011392.s004.docx]

Responses to Reviewers

We thank the reviewers for their comments and, in particular, their suggestions with regard to providing additional information in the Introduction concerning the immunopathology of *Wolbachia* in filarids. These comments, along with the changes suggested in the Discussion section, have led to improvement in the article as a whole.

Note that we have introduced continuous line numbering throughout the manuscript in order to facilitate references to the reviewers’ comments.

Line 1 (Title) Modified as requested by reviewer.

Line 15 (Key Words) Modified as requested by reviewer.

Line 22 (Abstract). No change required as the parasites in question are clearly the “parasitic filariae” mentioned in line 3. Moreover, the term “the parasites” is used at the end of line 7.

Line 27 (Abstract). Modified as requested by reviewer.

Line 57 (Author Summary). Modified as requested by reviewer.

Line 67 (Introduction). The vector species have been identified as requested by the reviewer in the comment in line 82

Line 73. Additional information provided as requested by reviewer.

Line 75. Additional information provided as requested by reviewer.

Line 82. As requested by the reviewer, the vector species have been identified (see line 67).

Line 83. No change required as “the two diseases” is sufficiently clear.

Line 87. The sentence has been modified for greater clarity.

Line 92. No change required as the current text reflects standard American usage.

Line 95. Reference added as requested by reviewer.

Line 98. Modified as requested by reviewer.

Line 114. Modified for greater clarity.

Line 115. No change required as the current wording is widely accepted in scientific publications.

Line 133 and following. Modified as requested by reviewer. Additional information has been added concerning the A·WOL consortium.

Lines 147, 148 and 150. No changes required as the current use of the hyphen is consistent with standard American usage.

Line 166. No change required as the current text reflects correct standard American usage.

Line 178. No change required as the current text reflects correct standard American usage.

Line 180. Modified as requested by reviewer.

Line 202. Modified as requested by reviewer.

Line 219. Modified as requested by reviewer.

Line 251. No change required as the current text reflects standard American usage.

Line 260. No change required as the current text reflects standard American usage.

Line 275. No change required as the current text reflects standard American usage.

Line 300. No change required as the current text is the MedDRA coding term for the AE and is used in Table 8.

Line 306. No change required as the current text reflects standard American usage.

Line 335 (Discussion). Modified as requested by reviewer.

Line 336. Modified as requested by reviewer.

Line 342. Modified as requested by reviewer.

Line 350. Modified as requested by reviewer.

Line 387. No change required as it is necessary to reiterate the findings in order to explain why changes were made to the study conduct and to introduce the resulting absence of further cases of elevated liver enzymes.

Line 407 (Acknowledgements). Typographical error corrected as requested by reviewer.

All Tables were inserted in to the body as per journal format.

Reference section. Three references have been added and the format of the references has been updated.
