# Supplementary material for: A Phase-I pharmacokinetic, safety and food-effect study on flubentylosin, a novel analog of Tylosin-A having potent anti-Wolbachia and antifilarial activity
Source: PLoS Negl Trop Dis. 2023 Jul 10;17(7):e0011392. doi: 10.1371/journal.pntd.0011392 (PMC10368248; doi:10.1371/journal.pntd.0011392)
Supplement: Attachment — Submitted filename: Reviewer comments R1_PLOS.doc [file pntd.0011392.s006.doc]

Dear Editor,

I have reviewed the manuscript PNTD-D-22-00302R1, entitled “A Phase-I pharmacokinetic, safety and food-effect study on flubentylosin, a novel analog of Tylosin-A having potent anti-*Wolbachia* and antifilarial activity” submitted to PLOS Neglected Tropical Diseases as a full-length article. I am pleased to inform that the R1 has considerably improved the paper. I have some minor corrections (pdf attached). Authors have carefully addressed the reviewer’s comments. Hence in my opinion this paper can be accepted for publication in PLOS Neglected Tropical Diseases.

Kind regards,

Ranju Manoj

_____________________________

Ranju Manoj, DVM, MVSc., PhD

Post-Doctoral Associate and Resident in Veterinary Parasitology

Department of Population Medicine and Diagnostics,

AHDC, Cornell University

Ithaca, New York

Tel +1 6465207636

e.mail: rm846@cornell.edu

e.mail: drrranjuvet@gmail.com

**COMMENTS TO THE AUTHORS**

I am pleased to inform that the R1 has considerably improved the paper. Authors have carefully addressed the reviewer’s comments. Hence in my opinion this paper can be accepted for publication in PLOS Neglected Tropical Diseases. Please see the pdf for some minor corrections.
